# Supplementary material for: The Contribution of the Minimal Promoter Element to the Activity of Synthetic Promoters Mediating CAR Expression in the Tumor Microenvironment
Source: Int J Mol Sci. 2022 Jul 4;23(13):7431. doi: 10.3390/ijms23137431 (PMC9266962; doi:10.3390/ijms23137431)
Supplement: Supplementary file 1 [file ijms-23-07431-s001.zip › ijms-1782453-supplementary.pptx]

## Slide 1
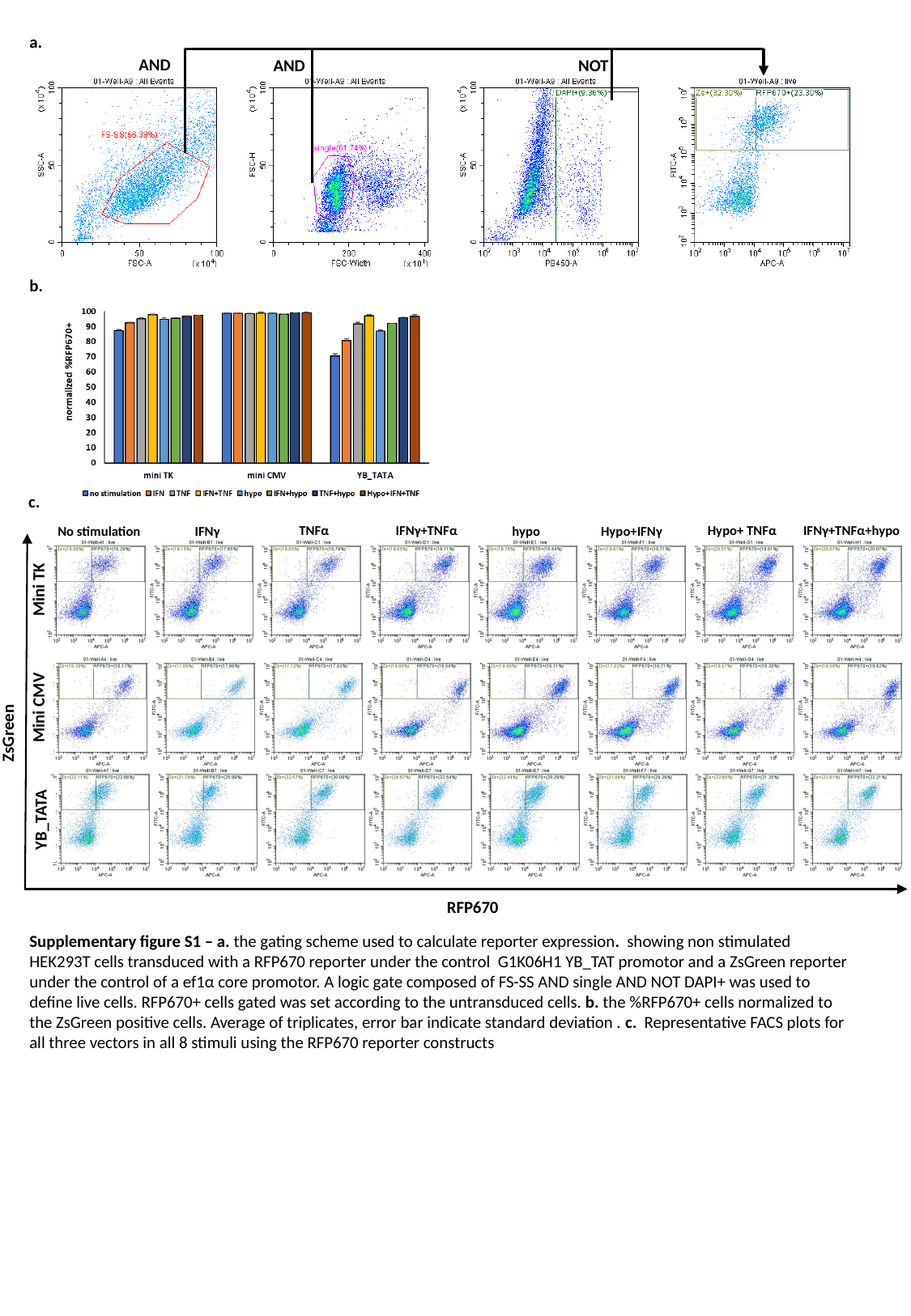

a.
AND
AND
NOT
b.
c.
IFNγ+TNFα+hypo
TNFα
IFNγ+TNFα
Hypo+ TNFα
No stimulation
IFNγ
Hypo+IFNγ
hypo
Mini TK
Mini CMV
ZsGreen
YB_TATA
RFP670
Supplementary figure S1 – a. the gating scheme used to calculate reporter expression. showing non stimulated HEK293T cells transduced with a RFP670 reporter under the control G1K06H1 YB_TAT promotor and a ZsGreen reporter under the control of a ef1α core promotor. A logic gate composed of FS-SS AND single AND NOT DAPI+ was used to define live cells. RFP670+ cells gated was set according to the untransduced cells. b. the %RFP670+ cells normalized to the ZsGreen positive cells. Average of triplicates, error bar indicate standard deviation . c. Representative FACS plots for all three vectors in all 8 stimuli using the RFP670 reporter constructs

## Slide 2
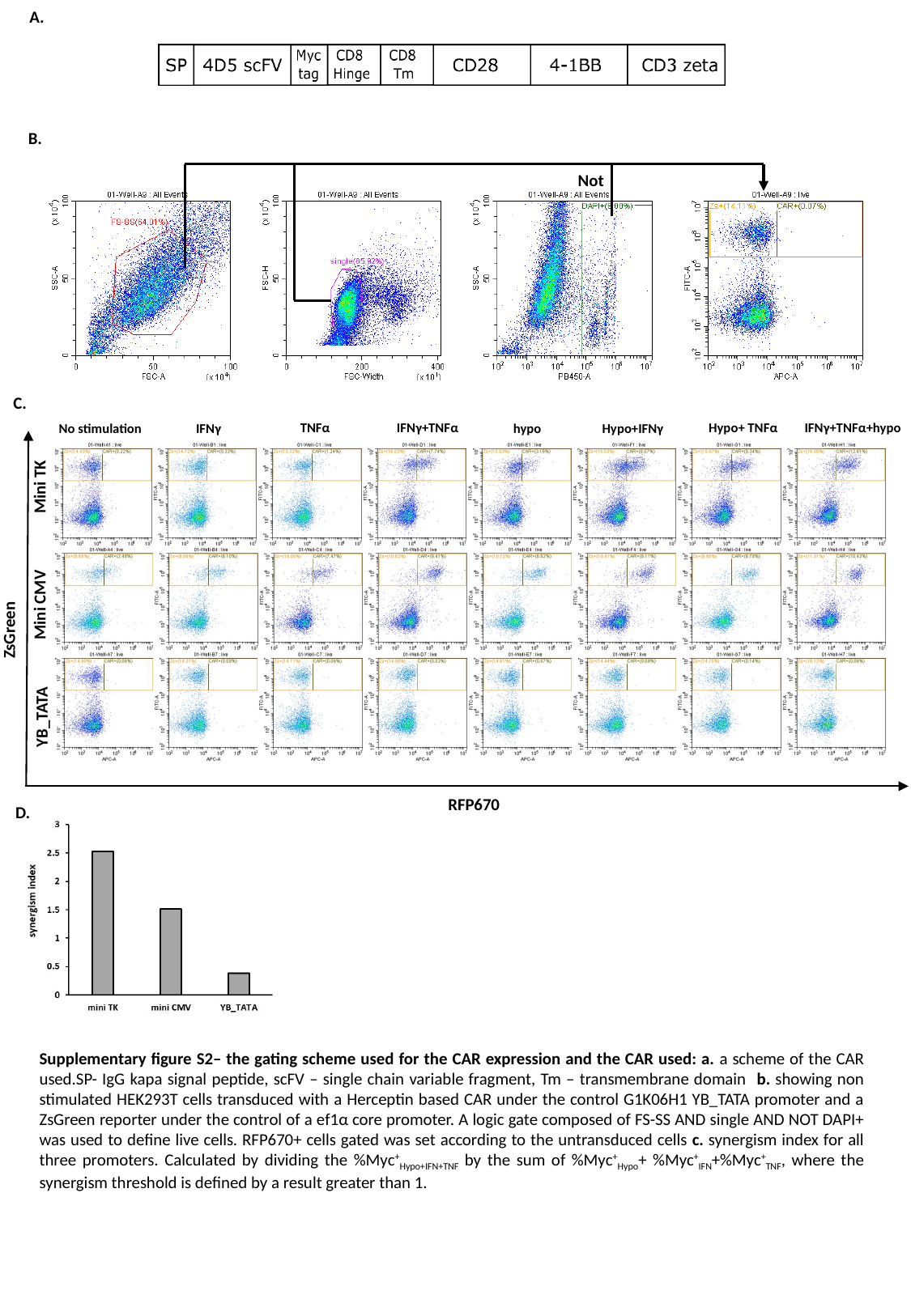

A.
B.
Not
C.
IFNγ+TNFα+hypo
TNFα
IFNγ+TNFα
Hypo+ TNFα
No stimulation
IFNγ
Hypo+IFNγ
hypo
Mini TK
Mini CMV
ZsGreen
YB_TATA
RFP670
D.
Supplementary figure S2– the gating scheme used for the CAR expression and the CAR used: a. a scheme of the CAR used.SP- IgG kapa signal peptide, scFV – single chain variable fragment, Tm – transmembrane domain b. showing non stimulated HEK293T cells transduced with a Herceptin based CAR under the control G1K06H1 YB_TATA promoter and a ZsGreen reporter under the control of a ef1α core promoter. A logic gate composed of FS-SS AND single AND NOT DAPI+ was used to define live cells. RFP670+ cells gated was set according to the untransduced cells c. synergism index for all three promoters. Calculated by dividing the %Myc+Hypo+IFN+TNF by the sum of %Myc+Hypo+ %Myc+IFN+%Myc+TNF, where the synergism threshold is defined by a result greater than 1.

## Slide 3
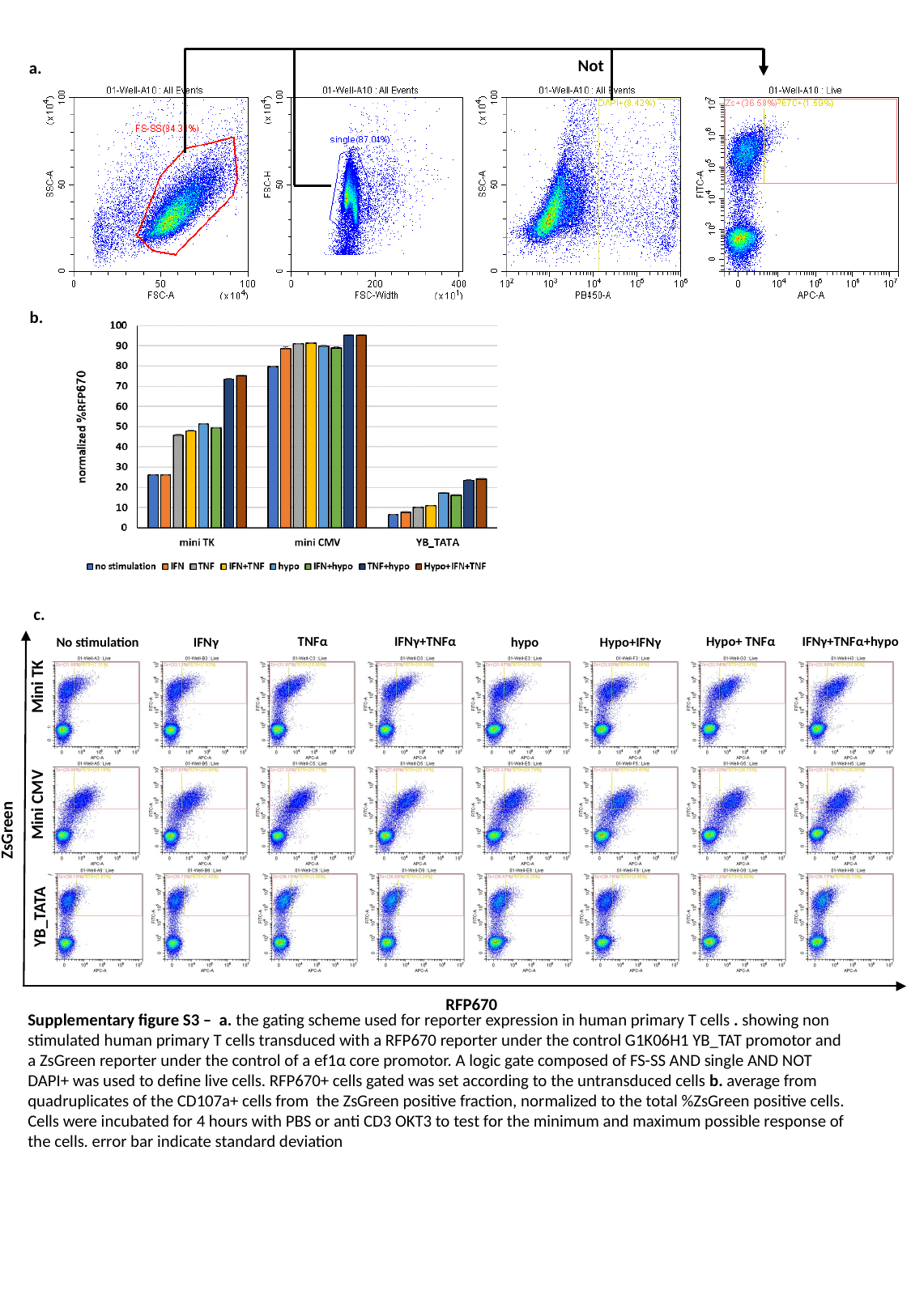

Not
a.
b.
c.
IFNγ+TNFα+hypo
TNFα
IFNγ+TNFα
Hypo+ TNFα
No stimulation
IFNγ
Hypo+IFNγ
hypo
Mini TK
Mini CMV
ZsGreen
YB_TATA
RFP670
Supplementary figure S3 – a. the gating scheme used for reporter expression in human primary T cells . showing non stimulated human primary T cells transduced with a RFP670 reporter under the control G1K06H1 YB_TAT promotor and a ZsGreen reporter under the control of a ef1α core promotor. A logic gate composed of FS-SS AND single AND NOT DAPI+ was used to define live cells. RFP670+ cells gated was set according to the untransduced cells b. average from quadruplicates of the CD107a+ cells from the ZsGreen positive fraction, normalized to the total %ZsGreen positive cells. Cells were incubated for 4 hours with PBS or anti CD3 OKT3 to test for the minimum and maximum possible response of the cells. error bar indicate standard deviation

## Slide 4
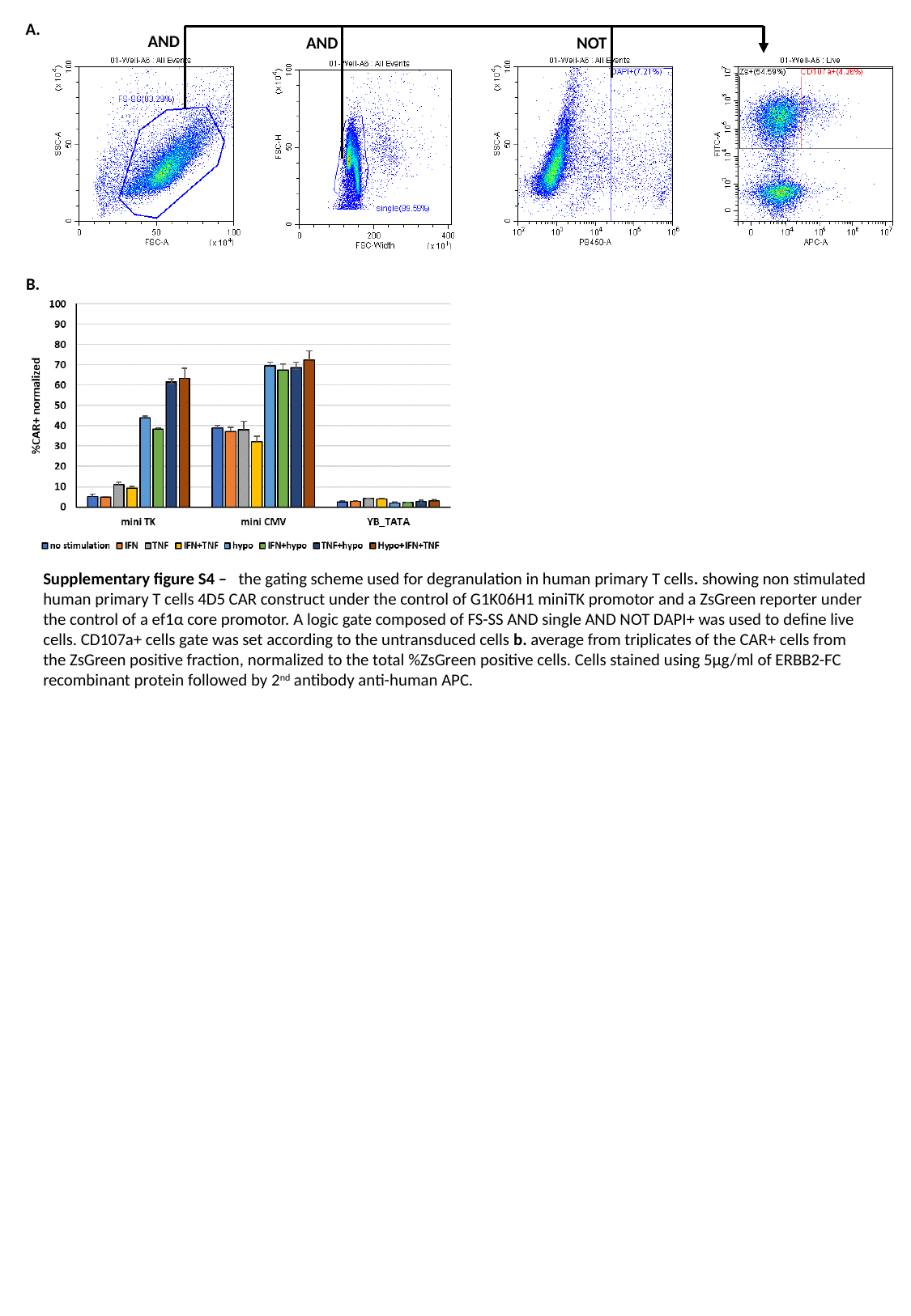

A.
AND
AND
NOT
B.
Supplementary figure S4 – the gating scheme used for degranulation in human primary T cells. showing non stimulated human primary T cells 4D5 CAR construct under the control of G1K06H1 miniTK promotor and a ZsGreen reporter under the control of a ef1α core promotor. A logic gate composed of FS-SS AND single AND NOT DAPI+ was used to define live cells. CD107a+ cells gate was set according to the untransduced cells b. average from triplicates of the CAR+ cells from the ZsGreen positive fraction, normalized to the total %ZsGreen positive cells. Cells stained using 5µg/ml of ERBB2-FC recombinant protein followed by 2nd antibody anti-human APC.

## Slide 5
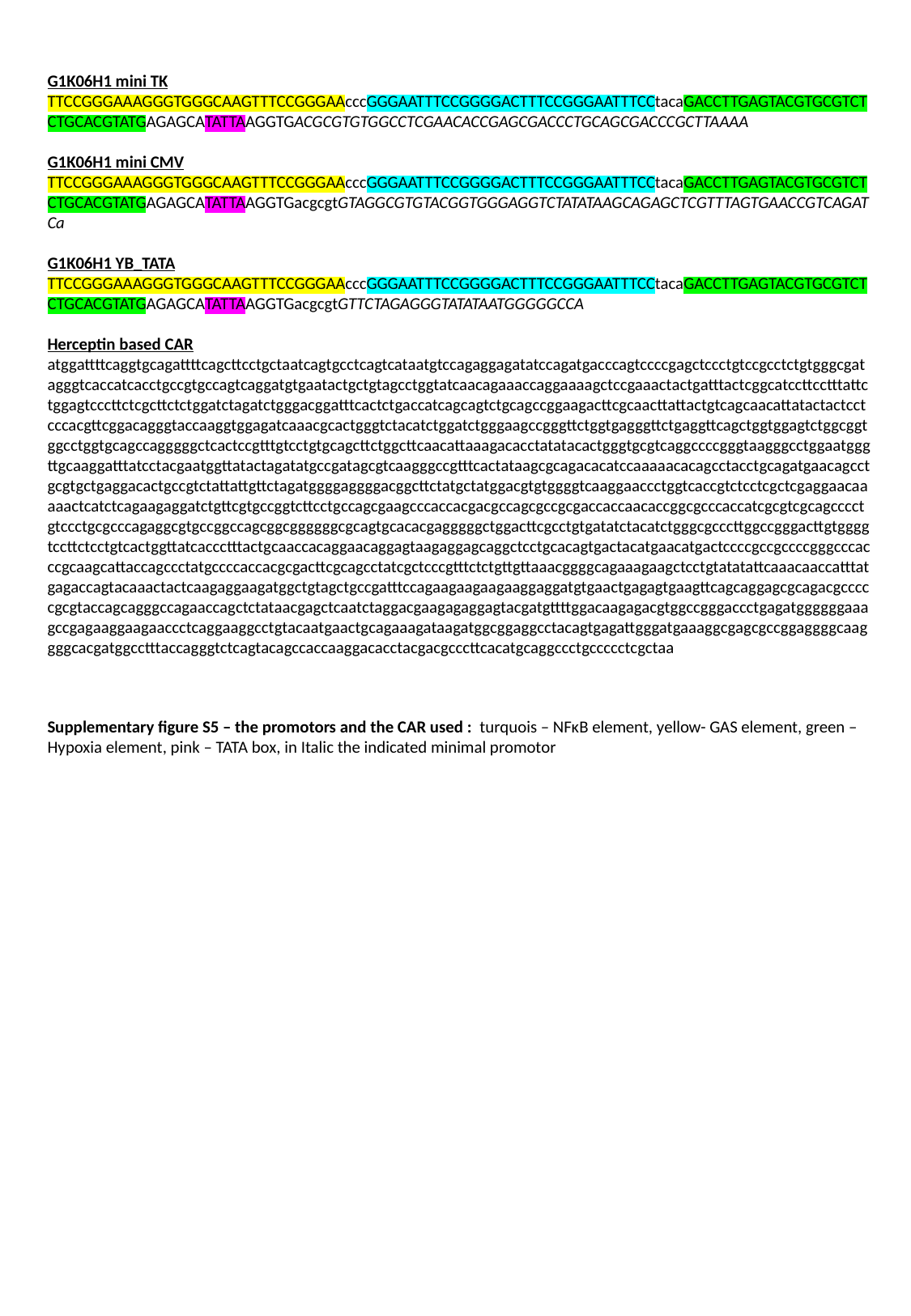

G1K06H1 mini TK
TTCCGGGAAAGGGTGGGCAAGTTTCCGGGAAcccGGGAATTTCCGGGGACTTTCCGGGAATTTCCtacaGACCTTGAGTACGTGCGTCTCTGCACGTATGAGAGCATATTAAGGTGACGCGTGTGGCCTCGAACACCGAGCGACCCTGCAGCGACCCGCTTAAAA
G1K06H1 mini CMV
TTCCGGGAAAGGGTGGGCAAGTTTCCGGGAAcccGGGAATTTCCGGGGACTTTCCGGGAATTTCCtacaGACCTTGAGTACGTGCGTCTCTGCACGTATGAGAGCATATTAAGGTGacgcgtGTAGGCGTGTACGGTGGGAGGTCTATATAAGCAGAGCTCGTTTAGTGAACCGTCAGATCa
G1K06H1 YB_TATA
TTCCGGGAAAGGGTGGGCAAGTTTCCGGGAAcccGGGAATTTCCGGGGACTTTCCGGGAATTTCCtacaGACCTTGAGTACGTGCGTCTCTGCACGTATGAGAGCATATTAAGGTGacgcgtGTTCTAGAGGGTATATAATGGGGGCCA
Herceptin based CAR
atggattttcaggtgcagattttcagcttcctgctaatcagtgcctcagtcataatgtccagaggagatatccagatgacccagtccccgagctccctgtccgcctctgtgggcgatagggtcaccatcacctgccgtgccagtcaggatgtgaatactgctgtagcctggtatcaacagaaaccaggaaaagctccgaaactactgatttactcggcatccttcctttattctggagtcccttctcgcttctctggatctagatctgggacggatttcactctgaccatcagcagtctgcagccggaagacttcgcaacttattactgtcagcaacattatactactcctcccacgttcggacagggtaccaaggtggagatcaaacgcactgggtctacatctggatctgggaagccgggttctggtgagggttctgaggttcagctggtggagtctggcggtggcctggtgcagccagggggctcactccgtttgtcctgtgcagcttctggcttcaacattaaagacacctatatacactgggtgcgtcaggccccgggtaagggcctggaatgggttgcaaggatttatcctacgaatggttatactagatatgccgatagcgtcaagggccgtttcactataagcgcagacacatccaaaaacacagcctacctgcagatgaacagcctgcgtgctgaggacactgccgtctattattgttctagatggggaggggacggcttctatgctatggacgtgtggggtcaaggaaccctggtcaccgtctcctcgctcgaggaacaaaaactcatctcagaagaggatctgttcgtgccggtcttcctgccagcgaagcccaccacgacgccagcgccgcgaccaccaacaccggcgcccaccatcgcgtcgcagcccctgtccctgcgcccagaggcgtgccggccagcggcggggggcgcagtgcacacgagggggctggacttcgcctgtgatatctacatctgggcgcccttggccgggacttgtggggtccttctcctgtcactggttatcaccctttactgcaaccacaggaacaggagtaagaggagcaggctcctgcacagtgactacatgaacatgactccccgccgccccgggcccacccgcaagcattaccagccctatgccccaccacgcgacttcgcagcctatcgctcccgtttctctgttgttaaacggggcagaaagaagctcctgtatatattcaaacaaccatttatgagaccagtacaaactactcaagaggaagatggctgtagctgccgatttccagaagaagaagaaggaggatgtgaactgagagtgaagttcagcaggagcgcagacgcccccgcgtaccagcagggccagaaccagctctataacgagctcaatctaggacgaagagaggagtacgatgttttggacaagagacgtggccgggaccctgagatggggggaaagccgagaaggaagaaccctcaggaaggcctgtacaatgaactgcagaaagataagatggcggaggcctacagtgagattgggatgaaaggcgagcgccggaggggcaaggggcacgatggcctttaccagggtctcagtacagccaccaaggacacctacgacgcccttcacatgcaggccctgccccctcgctaa
Supplementary figure S5 – the promotors and the CAR used : turquois – NFκB element, yellow- GAS element, green – Hypoxia element, pink – TATA box, in Italic the indicated minimal promotor
